# Molecular Dynamics Study of the Sintering Behavior and Mechanical Properties of Cu@Ag Core–Shell Nanoparticle Solder Paste

**DOI:** 10.3390/ma19081612

**Published:** 2026-04-17

**Authors:** Xuezhi Zhang, Jian Gao, Lanyu Zhang

**Affiliations:** State Key Laboratory of Precision Electronic Manufacturing Technology and Equipment, School of Electromechanical Engineering, Guangdong University of Technology, Guangzhou 510006, China; xuezhimail@163.com (X.Z.);

**Keywords:** Cu@Ag core–shell nanoparticle, molecular dynamics, sintering behavior, mechanical properties

## Abstract

Silver-coated copper (Cu@Ag) core–shell nanoparticles are promising interconnect materials for electronic packaging due to their high conductivity, oxidation resistance, and reduced use of precious metals. However, the key factors governing their sintering behavior and mechanical performance are not fully understood. In this study, molecular dynamics simulations were performed to examine the effects of sintering pressure (300–700 MPa), temperature (500–700 K), particle size, and silver shell thickness on atomic diffusion, microstructural evolution, and mechanical properties. Results show that higher pressure improves particle contact, accelerates densification, and strengthens interfacial bonding, with optimal performance achieved at 600–700 MPa. Elevated temperatures enhance atomic mobility, promoting neck growth and pore elimination, with the most active diffusion observed between 650 K and 700 K. Particle size and shell thickness also affect sintering: the Ag6Cu3 configuration exhibits the highest atomic mobility and a balanced combination of strength and ductility. Moderately thick silver shells facilitate surface diffusion and interfacial interdiffusion, while mechanisms such as the Kirkendall effect and local plastic relaxation reduce defect density, yielding stable sintered structures. These findings provide atomic-scale insights into the sintering mechanisms of Cu@Ag nanoparticle solder pastes and offer guidance for optimizing processing parameters in high-performance electronic packaging applications.

## 1. Introduction

With the rapid development of electronic information technology, electronic devices are continuously evolving toward higher integration, miniaturization, and higher power density, placing greater demands on semiconductor packaging interconnection technologies [1,2]. As a critical bonding material in the electronic packaging process, solder paste provides reliable mechanical and electrical connections as well as thermal conduction pathways between the chip and the substrate; its performance largely determines the stability and reliability of electronic devices [3]. Traditional soldering materials are gradually revealing certain limitations when faced with the demands of advanced electronic packaging; therefore, the development of novel interconnection materials with superior performance has become one of the key research focuses.

In recent years, due to the significant size effects and high surface energy of nanomaterials, nanoparticles can achieve rapid diffusion and densification at lower temperatures during the sintering process [4,5,6]. Consequently, nanoparticle solder pastes have gradually become an important research focus in the field of electronic packaging. Among the numerous nanoparticle solder paste material systems, copper nanoparticle solder paste and silver nanoparticle solder paste are the two most representative types [7]. Copper nanoparticle solder pastes offer advantages such as low cost and high electrical and thermal conductivity, and are considered an interconnect material with promising application prospects. However, copper nanoparticles are highly prone to oxidation in air; the resulting oxide layer hinders diffusion and bonding between particles, thereby affecting the solder paste’s sintering performance and the quality of the solder joints [8]. Consequently, the oxidation of copper nanoparticles has become one of the key factors limiting their application.

In contrast, silver nanoparticle solder pastes exhibit excellent oxidation resistance and good electrical conductivity, and can form relatively dense joint structures during sintering; consequently, they have attracted widespread attention in the field of electronic packaging [9]. However, due to the high cost of silver, their large-scale application still faces certain cost pressures [10]. To balance material performance with cost advantages, researchers have recently proposed preparing Cu@Ag core–shell nanoparticle solder pastes by coating the surface of copper nanoparticles with a silver shell [11,12]. This core–shell structure not only protects the copper core with the silver shell, thereby effectively suppressing the oxidation of copper particles, but also reduces material costs to some extent [13,14]. Consequently, it is considered an electronic packaging interconnect material with good application potential.

Currently, numerous scholars have conducted research on Cu@Ag nanoparticle solder pastes. Yang. et al. [15,16,17,18] have successfully synthesized Cu@Ag core–shell nanoparticles and conducted experimental analyses of their sintering behavior and microstructure; Wang. et al. [19] have investigated the effects of process parameters such as sintering temperature, pressure, and particle size on the structure and performance of solder joints; and Zhang. et al. [20,21] have explored the application potential of Cu@Ag nanoparticle solder pastes in electronic packaging interconnects. These studies provide an important experimental foundation for the application of Cu@Ag nanoparticle solder pastes and have advanced their development in the field of electronic packaging.

However, because the sintering of nanoparticles involves complex atomic diffusion and structural evolution, which typically occur on extremely short time scales and within very small spatial ranges, it is difficult to directly observe and accurately characterize these processes using traditional experimental methods [22,23]. In particular, at the atomic scale, there remains a lack of in-depth understanding of microscopic processes such as interfacial diffusion between particles, neck growth, and pore evolution [24,25]. Therefore, it is necessary to employ theoretical calculations and numerical simulation methods to further investigate the sintering mechanisms of nanoparticle-based solder pastes.

Molecular Dynamics (MD) simulation is a numerical simulation method based on classical mechanics that describes atomic motion by calculating interactions between atoms. This method enables the study of material structural evolution and dynamic behavior at the atomic scale, thereby offering significant advantages in the investigation of sintering mechanisms in nanomaterials. In recent years, studies have utilized molecular dynamics methods to analyze the sintering behavior of metal nanoparticle systems. For example, Gao et al. [26] have used MD simulations to investigate atomic diffusion behavior and neck growth mechanisms during the sintering process of nanoparticles; Kim et al. [5] have analyzed the effects of factors such as temperature and particle size on the sintering kinetics of nanoparticles; furthermore, Zhang et al. [27] have explored changes in the mechanical properties of nanoparticle structures after sintering. These studies provide important theoretical foundations for understanding the sintering process of nanoparticles.

Although some progress has been made in molecular dynamics studies of the sintering behavior of metal nanoparticles, most of the relevant work has focused on single-metal nanoparticle systems, and there remains relatively little research on silver-coated copper nanoparticle solder pastes with core–shell structures [28,29]. In particular, systematic studies on the sintering mechanisms and mechanical properties of Cu@Ag nanoparticle solder pastes at the atomic scale remain limited. Therefore, it is necessary to conduct a systematic investigation of the sintering behavior of Cu@Ag nanoparticle solder pastes using molecular dynamics methods to further elucidate the mechanisms of microstructural evolution and the patterns of property changes.

On the basis of the preceding analysis, a molecular dynamics model of Cu@Ag nanoparticle solder paste was established to further investigate its sintering behavior. Particular attention was given to the influence of key processing parameters, including sintering temperature, applied pressure, and particle size, on atomic diffusion and the evolution of microstructure during the sintering process. In addition, the mechanical response of the sintered structures was evaluated to clarify how different processing conditions affect the sintering mechanism and the resulting mechanical performance. The findings provide useful theoretical guidance for the design and optimization of Cu@Ag nanoparticle solder pastes for advanced electronic packaging.

## 2. Modeling and Methods

### 2.1. Basic Principles of Molecular Dynamics

Molecular Dynamics (MD) is a computational method based on classical mechanics theory, which simulates the motion behavior of atoms or molecules over a certain time scale through numerical calculations [6]. By solving the motion equations of all atoms in the system, this method can obtain the structural evolution and dynamic behavior of materials at the microscopic scale, and has been widely applied in fields such as materials science, nanotechnology, and condensed matter physics.

In molecular dynamics simulations, the motion of each atom in the system follows Newton’s second law, which can be expressed in its basic form as:

(1) $$F_{i}=m_{i}a_{i}$$

where $F_{i}$ represents the resultant force acting on the i-th atom, $m_{i}$ is the atomic mass, and $a_{i}$ is the acceleration of the atom. The force exerted on an atom primarily originates from the interaction potential function between atoms in the system. By calculating the interaction potential energy between atoms and evaluating its gradient, the force acting on the atom can be obtained, which further allows for the solution of the atomic motion trajectory.

In practical calculations, molecular dynamics methods typically employ numerical integration algorithms to solve the equations of motion for atoms, with commonly used algorithms including the Verlet algorithm and the Velocity-Verlet algorithm. Through iterative computations, the atomic positions, velocities, and energy variations in the system at different time steps can be obtained, thereby enabling the simulation of microstructural evolution in materials. As molecular dynamics methods can directly characterize changes in the internal structure of materials at the atomic scale, they demonstrate significant advantages in studying atomic diffusion, interfacial bonding, and structural evolution during the sintering process of nanoparticles. In this study, molecular dynamics simulations were conducted using the Large-scale Atomic/Molecular Massively Parallel Simulator (LAMMPS, version stable_22Jul2025_update3; available from https://www.lammps.org/ (accessed on 1 March 2026)), and the atomic data obtained from the simulations were processed and analyzed using the Open Visualization Tool (OVITO, version 3.13.0; available from https://www.ovito.org/ (accessed on 1 March 2026)).

### 2.2. Interatomic Potential Function

In molecular dynamics simulations, the interactions between atoms are typically described by potential functions, and the choice of potential function significantly influences the accuracy of the simulation results. For metallic material systems, the Embedded Atom Method (EAM) is a widely used many-body potential function that effectively captures the interactions between metal atoms.

The EAM potential function posits that the total energy of an atom consists of two components: one is the embedding energy arising from embedding the atom into the surrounding electron density, and the other is the pair potential interaction between atoms. The total energy of the system can be expressed as

(2) $$E=\sum_{i}F\left(\rho_{i}\right)+\frac{1}{2}\sum_{i\neq j}\varphi \left(r_{ij}\right)$$

where $F\left(\rho_{i}\right)$ represents the embedding energy function, $\rho_{i}$ is the local electron density at the site of atom i, $\varphi \left(r_{ij}\right)$ is the pair potential function between atom i and atom j, and $r_{ij}$ denotes the distance between the two atoms.

Since both silver (Ag) and copper (Cu) are face-centered cubic (FCC) metals, the EAM potential function is well-suited to describe their crystal structures, elastic properties, and defect behaviors. Therefore, in this study, the EAM potential function parameterized for the Ag–Cu alloy system was employed to characterize the interatomic interactions [30]. This potential has been widely validated in previous studies for accurately reproducing key properties, including lattice constants, elastic constants, surface energies, and diffusion behavior of Cu, Ag, and their alloys.

### 2.3. Molecular Dynamics Model

**Figure 1:**
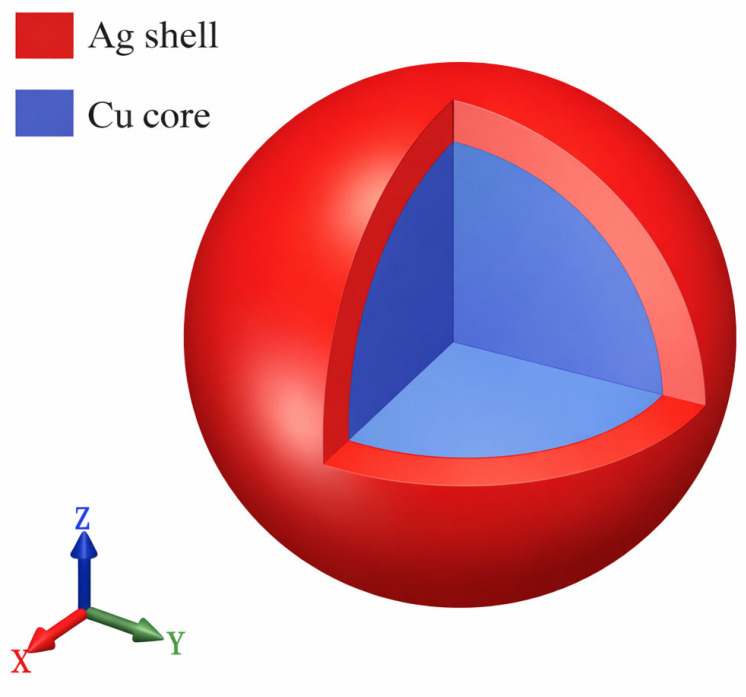
Isometric projection of the upper and lower axes of a Cu@Ag core–shell nanoparticles model.

**Figure 2:**
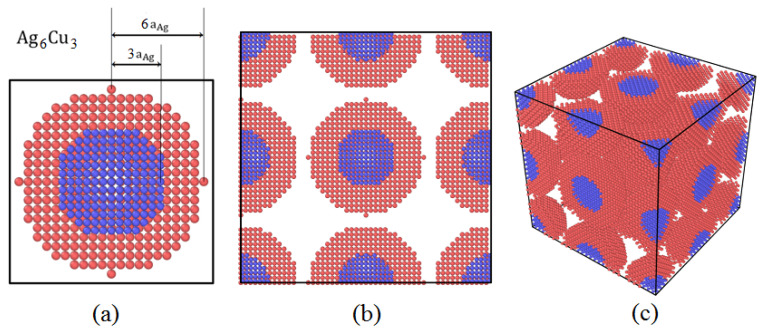
Model of the Ag6Cu3 core–shell structure. (**a**) Individual Cu@Ag core–shell nanoparticle. (**b**) Frontal view of the initial structure of the sintered model. (**c**) 3D view.

To investigate the sintering behavior of silver-coated copper nanoparticle paste, this study established a model of Ag@Cu core–shell nanoparticles. Given the complexity of realistic sintering models, appropriate simplifications were made to the simulated system to facilitate computational efficiency. A schematic isometric diagram of the Cu-Ag core–shell model is shown in Figure 1, where copper nanoparticles serve as the core, coated with a layer of silver atoms to form the core–shell structure. The actual simulation system is illustrated in Figure 2, where each simulation box contains eight Cu-Ag core–shell nanoparticles. Specifically, one-eighth of a particle is located at each of the eight corners, one-half of a particle is positioned at the centers of the six faces, one-quarter of a particle is situated at the centers of the twelve edges, and a whole particle is placed at the center of the simulation box. The separation distance between adjacent Cu-Ag core–shell nanoparticles was set to 4.085 Å (the lattice constant of Ag, aAg), which not only prevents contact during relaxation but also ensures that the particles remain within the cutoff radius and maintain interactions.

**Figure 3:**
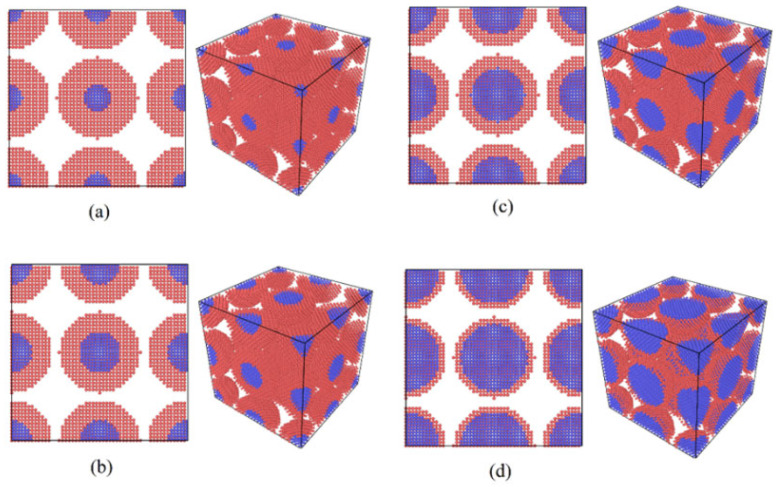
Models with different silver shell thicknesses: (**a**) Ag6Cu2, (**b**) Ag6Cu3, (**c**) Ag6Cu4, and (**d**) Ag6Cu5.

To investigate the influence of silver shell thickness on the sintering of Cu-Ag core–shell nanoparticles, four additional sintering models—Ag6Cu1, Ag6Cu2, Ag6Cu4, and Ag6Cu5—were established based on the previously constructed Ag6Cu3 sintering model. The sintering models with varying silver shell thicknesses are illustrated in Figure 3, and the atomic numbers and corresponding proportions for each individual particle are summarized in Table 1.

**Figure 4:**
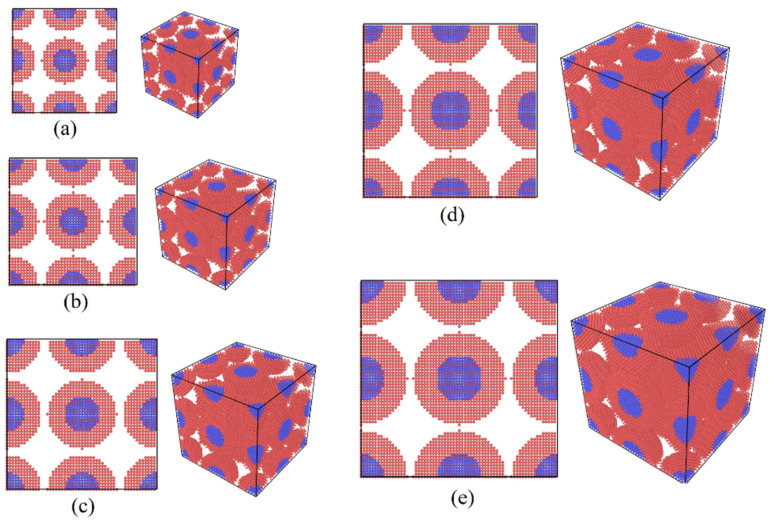
Models with different particle sizes: (**a**) Ag4Cu2, (**b**) Ag5Cu2.5, (**c**) Ag6Cu3, (**d**) Ag7Cu3.5, and (**e**) Ag8Cu4.

To investigate the effect of particle size on the sintering behavior of Cu–Ag core–shell nanoparticles, four additional sintering models—Ag4Cu2, Ag5Cu2.5, Ag7Cu3.5, and Ag8Cu4—were constructed based on the previously established Ag6Cu3 model. These models with varying particle sizes are illustrated in Figure 4, all of which maintain identical silver shell thickness and copper core radius.

### 2.4. Simulation Parameters and Analytical Methods

In this study, five distinct sintering temperatures (500, 550, 600, 650, and 700 K) and five different sintering pressures (300, 400, 500, 600, and 700 MPa) were applied to the Ag6Cu3 sintering model to investigate their respective effects on the mechanical properties of sintered Cu–Ag core–shell nanoparticles. The pressure was applied using an isothermal–isobaric (NPT) ensemble with a barostat, allowing the simulation box to shrink dynamically under the prescribed external pressure during the sintering process. A time step of 1 fs was employed in the molecular dynamics simulations to ensure numerical stability and computational accuracy. Periodic boundary conditions were adopted throughout the simulation to minimize boundary effects on the results. In the present study, periodic boundary conditions were applied, and the simulation cell size was carefully selected to minimize finite-size effects. While explicit finite-size corrections were not applied, we ensured that the system dimensions were sufficiently large to capture the essential physical behavior, particularly for diffusion and mechanical response. Uniaxial tension was applied to the sintered structure along the X-axis at a strain rate of 0.01/ps. The strain rate of 0.01/ps, while high compared to experimental conditions, is commonly used in molecular dynamics simulations due to computational time scale limitations. The strain rate of 0.01/ps adopted in this study is primarily dictated by the inherent time-scale limitations of molecular dynamics simulations, as significantly lower strain rates would be computationally infeasible. Although such a high strain rate may lead to an overestimation of the absolute mechanical properties, it does not alter the relative trends observed among different systems; therefore, the results remain meaningful for comparative analysis of their mechanical behavior. Taking a sintering temperature of 600 K and a sintering pressure of 500 MPa as an example, the detailed sintering and tensile procedures are summarized in Table 2. In this study, the selected parameter range (e.g., temperature, pressure, and core–shell structure) was chosen based on typical processing conditions reported for nano-solder pastes in electronic packaging applications, ensuring both physical relevance and computational feasibility. The temperature and pressure were controlled using a Nose–Hoover thermostat. The Nose–Hoover barostat was employed due to its proven capability to ensure stable and physically consistent pressure control, making it well-suited for maintaining reliable thermodynamic conditions during both the sintering and subsequent equilibration processes.

To analyze the microstructural evolution of the nanoparticle system during sintering, various structural analysis methods were employed in this study, including Common Neighbor Analysis (CNA) and Mean Square Displacement (MSD). The diffusion coefficient can be extracted from the MSD curve based on the Einstein relation. In practice, one selects the linear region of the MSD–time curve (typically after the initial ballistic regime) and performs a linear fit to obtain the slope. In a three-dimensional system, the diffusion coefficient can be obtained by dividing the slope by 6. This approach ensures that the extracted diffusion coefficient reflects steady-state diffusive behavior rather than transient dynamics. The CNA method is primarily used to identify crystal structure types within the system, such as FCC, HCP, and disordered structures, while the MSD method is utilized to characterize the atomic diffusion behavior during the sintering process. The defining formula is as follows:

(3) $$\langle d^{2}\rangle ={\left\{\left[r\left(t_{0}+\tau \right)-r_{com}\left(t_{0}+\tau \right)\right]-\left[r\left(t_{0}\right)-r_{com}\left(t_{0}\right)\right]\right\}}^{2}$$

where $t_{0}$ is the initial time, τ is the observation time, $r\left(t_{0}\right)$ is the coordinate of the atom at time $t_{0}$, and $r_{com}\left(t_{0}\right)$ is the coordinate of the particle’s center of mass at time $t_{0}$. $r\left(t_{0}+\tau \right)$ is the atomic coordinate of the atom at time τ, and $r_{com}\left(t_{0}+\tau \right)$ is the coordinate of the particle’s center of mass at time τ.

Densification is a parameter characterizing the sintering performance in multi-particle models. The densification at time t is equal to the volume change in the simulation box divided by its initial volume. It can be expressed as follows:

(4) $$\delta =\frac{V_{0}-V_{t}}{V_{0}}$$

## 3. Results and Discussion

### 3.1. Analysis of the Sintering Process Using Common Neighbor Analysis (CNA)

**Figure 5:**
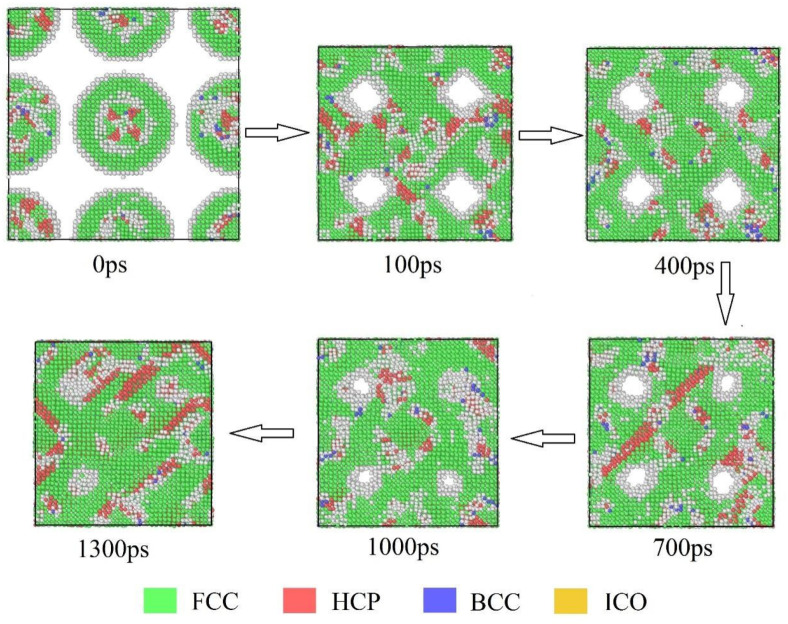
CNA of the sintering process of Ag6Cu3 core–shell nanoparticle solder paste.

As shown in Figure 5, the Ag@Cu core–shell nanoparticle solder paste exhibits pronounced microstructural evolution during the sintering process. At the initial state (0 ps), the particles are regularly arranged, with the copper cores encapsulated by silver shells. The structure is dominated by face-centered cubic (FCC, green) atoms, while a small number of hexagonal close-packed (HCP, red) and body-centered cubic (BCC, blue) defects are present at particle boundaries or surfaces. By 100 ps, atomic necks gradually form between particles. The FCC structure remains predominant, but HCP regions begin to appear along the necks, indicating local atomic rearrangement and lattice mismatch.

When the sintering time reaches 400 ps, the interparticle necks become noticeably larger and the connections between particles become more compact. The fraction of FCC atoms further increases, suggesting progressive structural stabilization. Meanwhile, HCP atoms appear in narrow bands near the neck regions, which is typically related to stacking faults induced by local stress during structural rearrangement. Between 700 ps and 1000 ps, a continuous interparticle network gradually develops. During this stage, atomic diffusion remains active along the neck regions and interfaces. Some atoms that initially belong to disordered configurations transform into FCC structures, indicating a tendency toward structural ordering. BCC atoms remain relatively scarce throughout this stage, implying that high-energy lattice configurations are not energetically favored in the sintering process. At approximately 1300 ps, the sintered structure becomes significantly more compact. The system reaches a relatively stable, partially or fully densified configuration depending on the applied pressure, with reduced porosity and enhanced particle contact. The FCC phase occupies most of the system, while the previously observed HCP bands decrease in both size and quantity. These observations indicate that the system approaches a relatively stable crystalline configuration as sintering proceeds.

From a mechanistic perspective, the gradual increase in FCC structures during sintering is closely related to atomic self-diffusion and lattice reorganization. At the early stage, differences in surface energy and interfacial energy provide the driving force for atomic migration, leading to the formation of interparticle necks. The appearance of HCP regions is mainly associated with local lattice distortion and dislocation activity, which helps accommodate internal stress generated during particle coalescence. As diffusion continues, many of these metastable configurations progressively transform into the energetically favorable FCC structure. The limited presence of BCC atoms suggests that high-energy or amorphous configurations are relatively unstable under the simulated sintering conditions and tend to convert into FCC structures over time. In addition, the Ag@Cu core–shell architecture influences atomic transport pathways and interfacial bonding behavior. This structural feature promotes the formation of a dense FCC-dominated network while retaining a small fraction of HCP regions that act as local stress accommodation sites, thereby contributing to the structural stability of the sintered body. The CNA results reveal a progressive structural transition during sintering, characterized by the transformation from an initially FCC-dominated configuration to a transient state with pronounced HCP-rich regions, and ultimately to a densely packed FCC matrix with reduced defect content. The emergence of HCP structures along interparticle necks indicates the formation of stacking faults and local lattice distortions, which are commonly associated with stress accommodation and interface-driven plasticity in nanoscale systems. As sintering proceeds, these metastable HCP regions gradually transform into the energetically favorable FCC phase, consistent with classical thermodynamic predictions and atomistic simulations of defect annealing driven by surface energy minimization and diffusion [31,32]. This evolution reflects a diffusion-controlled densification mechanism, where the reduction in high-energy interfaces and the relaxation of lattice mismatch collectively promote structural ordering and phase stability.

### 3.2. Effect of Different Variables on the Mechanical Properties of the Sintered Body

#### 3.2.1. Sintering Pressure

**Figure 6:**
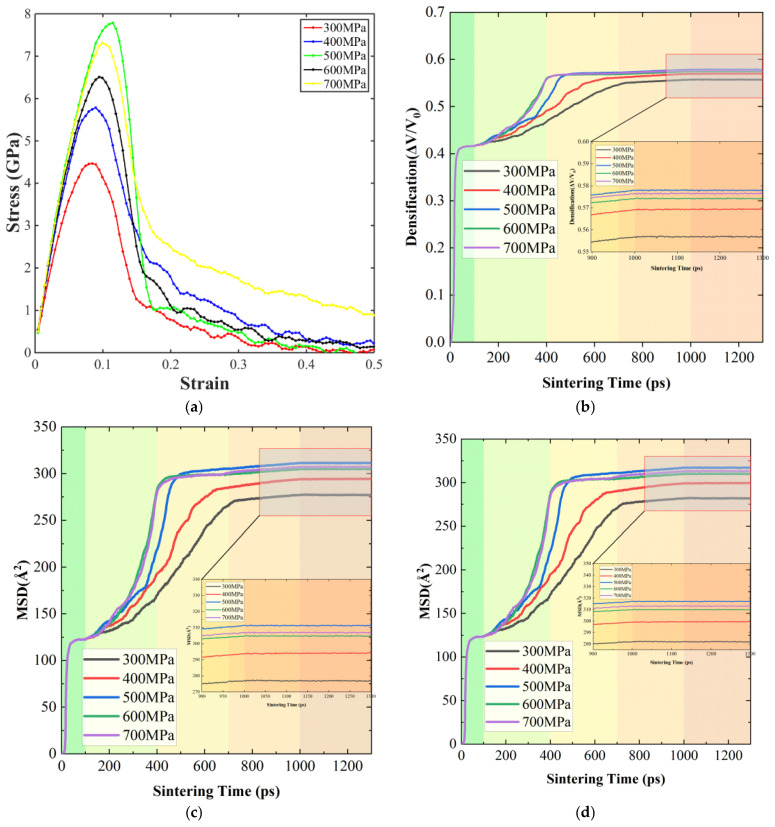
Effect of different sintering pressures (300, 400, 500, 600, and 700 MPa) on the mechanical properties of the sintered bodies: (**a**) Uniaxial tensile stress–strain curves; (**b**) densification curves during the sintering process; (**c**) MSD of all atoms during sintering (MSD_All); (**d**) MSD of Ag atoms during sintering (MSD_Ag).

The influence of sintering pressure on the mechanical response and diffusion behavior of Ag@Cu nanoparticle sintered bodies is summarized in Figure 6, which includes the stress–strain curves, densification evolution, and the mean square displacement (MSD) of atoms under pressures ranging from 300 to 700 MPa.

The stress–strain curves were obtained after the sintering and pressure-assisted densification stage. Once the hot-pressing sintering of the simulation system is completed, the external pressure will be removed. The sintered body obtained after hot pressing sintering is subjected to uniaxial tension to observe its mechanical properties. In this study, the stress–strain curve was obtained using LAMMPS by applying uniaxial deformation at a constant strain rate via the “fix deform” command, where the strain was calculated from the relative change in the simulation box length along the loading direction, and the corresponding stress was derived from the virial-based pressure tensor normalized by the system volume; the stress–strain relationship was subsequently constructed from the recorded data during the deformation process.

The stress–strain curves in Figure 6a show that the mechanical strength of the sintered structures improves progressively as the applied pressure increases. Under a relatively low pressure of 300 MPa, the sample exhibits a small peak stress and an early stress drop, suggesting weak bonding between particles and the presence of residual pores or structural defects. When the pressure is increased to 400 MPa and 500 MPa, both the peak stress and the elastic modulus rise noticeably. This behavior indicates that particle contacts become more effective and the structural continuity of the sintered body improves. Further increasing the pressure to 600 MPa and 700 MPa leads to the highest peak stress values, implying stronger interfacial bonding and improved load-bearing capability. Nevertheless, a reduction in stress is still observed at large strains, which is mainly related to plastic deformation processes such as dislocation motion, grain boundary migration, and local structural rearrangement.

The densification evolution during sintering is presented in Figure 6b. In all pressure conditions, the relative density increases gradually with time and approaches a stable value after roughly 400–600 ps. This stage corresponds to rapid neck formation and atomic diffusion between neighboring particles. Higher sintering pressure noticeably accelerates the densification process. Increased pressure improves particle contact and enlarges the effective diffusion pathways, which reduces the energy barrier for atomic migration. As a result, interfacial bonding and structural rearrangement occur more efficiently, enabling the system to reach a dense and stable configuration within a shorter time.

The diffusion behavior is further illustrated by the MSD curves of the entire atomic system and the Ag atoms, as shown in Figure 6c,d. In general, the MSD increases with simulation time, reflecting continuous atomic migration during the sintering process. A rapid increase in MSD occurs at around 400 ps, corresponding to the stage of intensive neck growth between particles. With increasing pressure, the slope of the MSD curves becomes steeper, indicating that atomic mobility is enhanced. This effect can be attributed to the larger contact area and the stronger stress gradients that develop at particle interfaces under high pressure, both of which facilitate atomic movement along interfaces and grain boundaries. The MSD behavior of Ag atoms follows a trend similar to that of the overall system but exhibits slightly larger values, suggesting that atoms in the silver shell possess relatively higher mobility and play an active role in promoting interparticle bonding.

Overall, sintering pressure strongly influences particle contact, atomic diffusion, and structural densification in Ag@Cu nanoparticle solder paste. Increasing the pressure within an appropriate range enhances interfacial bonding and accelerates densification, which ultimately leads to improved mechanical performance of the sintered structure.

#### 3.2.2. Sintering Temperature

**Figure 7:**
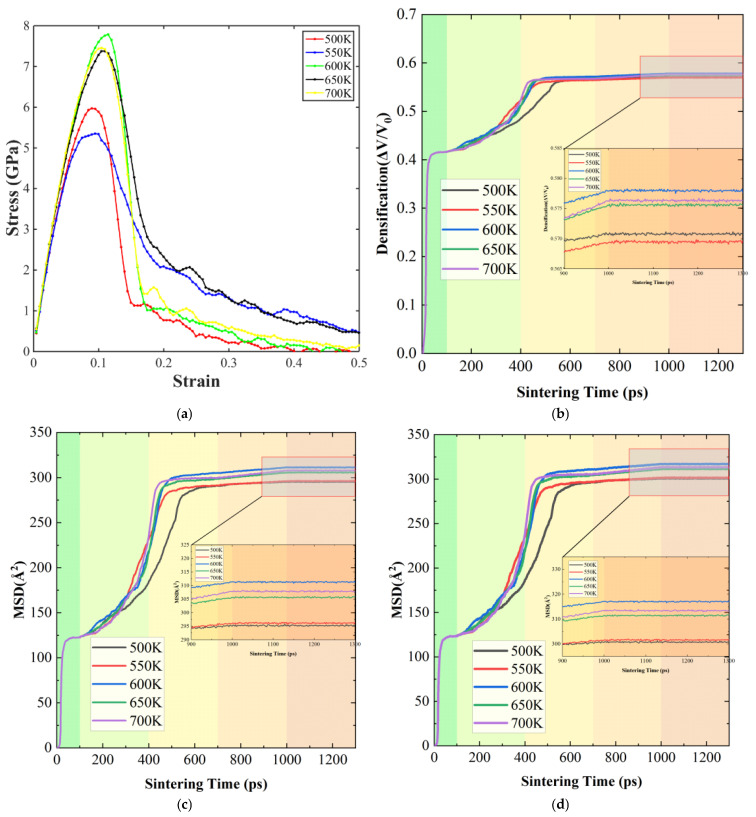
Effect of different sintering temperatures (500, 550, 600, 650, and 700 K) on the mechanical properties of the sintered bodies: (**a**) Uniaxial tensile stress–strain curves; (**b**) densification curves during the sintering process; (**c**) MSD of all atoms during sintering (MSD_All); (**d**) MSD of Ag atoms during sintering (MSD_Ag).

The effect of sintering temperature on the mechanical properties and atomic diffusion of Ag@Cu nanoparticle sintered bodies is shown in Figure 7. The figure includes the stress–strain curves, densification evolution, and MSD variations for temperatures ranging from 500 K to 700 K. The stress–strain curves displayed in Figure 7a reveal a clear dependence of mechanical performance on sintering temperature. At relatively low temperatures of 500 K and 550 K, the peak stress remains small and a noticeable stress drop appears at low strain levels. This behavior indicates that atomic diffusion is insufficient under these conditions, resulting in incomplete bonding between particles and the persistence of pores or structural imperfections. When the temperature is increased to 600 K and 650 K, the peak stress rises significantly and the elastic portion of the curve becomes steeper. These changes suggest that stronger particle connections are established and the sintered structure becomes more compact. At 700 K, the highest peak stress is obtained, indicating that elevated temperature effectively enhances interfacial bonding and improves the load-bearing capability of the material.

Figure 7b illustrates the densification process at different temperatures. During the initial stage of sintering (approximately 0–200 ps), the relative density evolves in a similar manner for all temperature conditions. As the process continues into the intermediate stage (around 200–500 ps), densification proceeds rapidly due to the formation and growth of interparticle necks. Increasing the temperature accelerates this process because stronger thermal vibration promotes atomic migration toward particle contact regions. Enhanced diffusion facilitates the elimination of pores and contributes to the formation of a more compact structure.

Figure 7c,d show the evolution of the mean square displacement (MSD) for all atoms and for Ag atoms specifically. Across all temperatures, MSD generally increases over time, with a pronounced rise around 400 ps, corresponding to rapid formation of interparticle necks. As the sintering temperature rises, the MSD curves shift upward, indicating that higher temperatures enhance atomic diffusion throughout the system. The MSD of Ag atoms follows a trend similar to that of the entire atomic ensemble but remains slightly higher, reflecting the greater mobility of silver atoms in the shell layer. This increased atomic movement at particle interfaces promotes interparticle bonding and enables structural rearrangements during sintering.

Overall, higher sintering temperatures accelerate atomic migration, strengthen interparticle connections, and support faster densification, collectively contributing to improved mechanical properties of the sintered bodies.

#### 3.2.3. Nanoparticle Diameter

**Figure 8:**
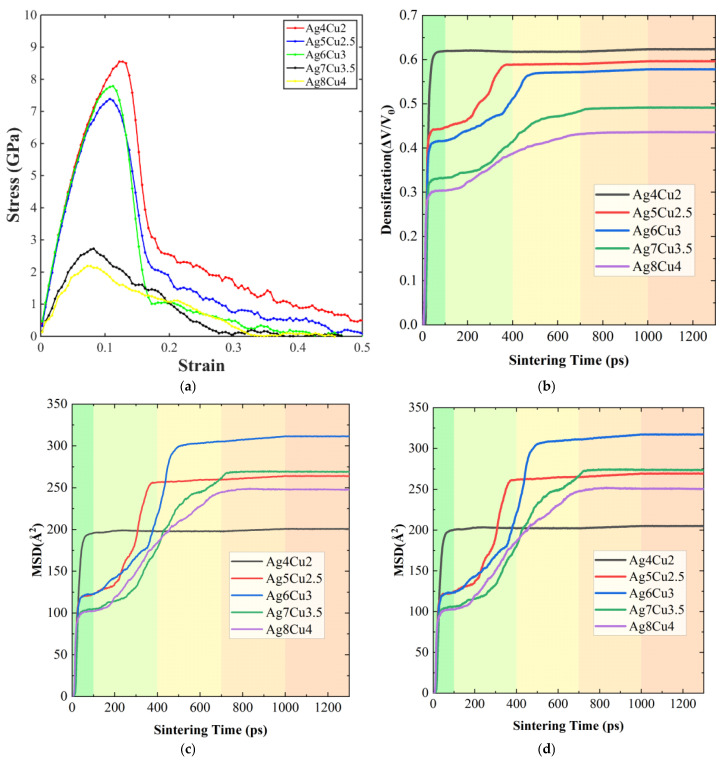
Effect of different nanoparticle diameters (Ag4Cu2, Ag5Cu2.5, Ag6Cu3, Ag7Cu3.5, and Ag8Cu4) on the mechanical properties of the sintered bodies: (**a**) Uniaxial tensile stress–strain curves; (**b**) densification curves during the sintering process; (**c**) MSD of all atoms during sintering (MSD_All); (**d**) MSD of Ag atoms during sintering (MSD_Ag).

Figure 8 compares the influence of nanoparticle diameter (Ag4Cu2 to Ag8Cu4) on the mechanical behavior and sintering characteristics of the simulated structures, including stress–strain response, densification evolution, and atomic diffusion activity.

The tensile stress–strain curves in Figure 8a indicate that nanoparticle size has a noticeable impact on the mechanical performance of the sintered bodies. As particle diameter increases, both the tensile strength and fracture strain show a non-linear trend, first increasing and then decreasing. Among the investigated configurations, the Ag6Cu3 structure exhibits the most favorable mechanical response, characterized by the highest tensile strength and a pronounced plastic deformation stage. In contrast, the smaller Ag4Cu2 particles display relatively limited strength and ductility, despite their higher surface energy. The largest particles, Ag8Cu4, show a decline in mechanical performance, suggesting that excessively large particles reduce the effectiveness of interparticle bonding during sintering. These observations imply that an appropriate particle size range is required to achieve optimal structural integrity.

The densification behavior under different particle sizes is presented in Figure 8b. At the early stage of sintering, all systems experience rapid volume shrinkage as neighboring particles establish initial contact and necks begin to form. As sintering continues, the densification rate gradually decreases and eventually approaches a stable value. Among the samples, the Ag6Cu3 structure shows the fastest densification rate and the largest final volume contraction, indicating the formation of a more compact structure. By comparison, the Ag4Cu2 and Ag8Cu4 systems reach lower final densities, suggesting that residual pores or structural heterogeneity remain within these sintered bodies.

Further insight into the mechanism can be obtained from the mean square displacement (MSD) curves shown in Figure 8c,d, which represent the diffusion behavior of all atoms and Ag atoms during sintering. MSD values increase continuously with simulation time, reflecting the progressive migration of atoms within the system. The Ag6Cu3 configuration exhibits consistently higher MSD values than the other structures, indicating stronger atomic mobility and more active sintering behavior. The MSD curves of Ag atoms follow a similar trend to that of the entire atomic system but maintain slightly larger magnitudes, suggesting that atoms in the silver shell participate actively in diffusion and facilitate particle rearrangement and interfacial bonding.

The observed size dependence can be explained from the perspective of surface energy and diffusion driving force. Smaller nanoparticles, such as Ag4Cu2, possess higher surface energy, which theoretically favors sintering. However, excessively small particles tend to agglomerate rapidly at the early stage, resulting in locally dense but globally heterogeneous structures that hinder subsequent densification. In contrast, larger particles such as Ag8Cu4 have relatively lower surface energy and reduced atomic mobility, which weakens the driving force for interfacial bonding. Particles with intermediate dimensions, represented by the Ag6Cu3 configuration, provide a balance between these two effects. This size range maintains sufficient surface energy to drive diffusion while preserving structural stability, allowing more uniform atomic transport and densification and ultimately producing better mechanical performance.

Overall, nanoparticle diameter strongly influences diffusion activity, densification behavior, and the final mechanical properties of the sintered structures. The intermediate particle size represented by Ag6Cu3 provides the most favorable combination of diffusion capability and structural stability, resulting in improved strength and ductility of the sintered body.

#### 3.2.4. Silver Shell Thickness

**Figure 9:**
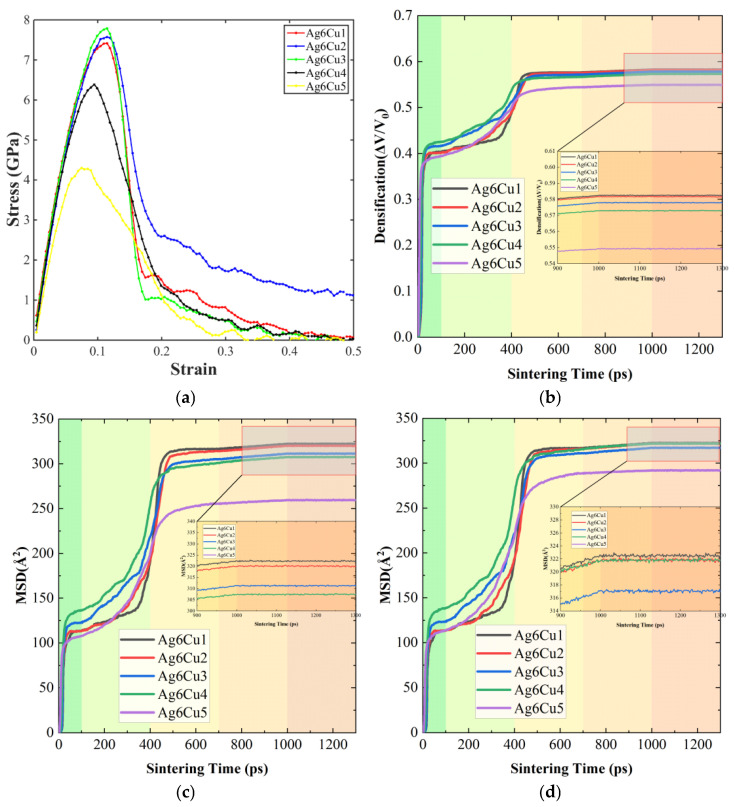
Effect of different silver shell thicknesses (Ag6Cu1, Ag6Cu2, Ag6Cu3, Ag6Cu4, and Ag6Cu5) on the mechanical properties of the sintered bodies: (**a**) Uniaxial tensile stress–strain curves; (**b**) densification curves during the sintering process; (**c**) MSD of all atoms during sintering (MSD_All); (**d**) MSD of Ag atoms during sintering (MSD_Ag).

The effect of silver shell thickness on the mechanical response and structural evolution of the core–shell nanoparticles is illustrated in Figure 9. In this section, particles are denoted as Ag6Cux, where the copper core diameter is fixed at 6 nm while the silver shell thickness increases with x.

The stress–strain curves shown in Figure 9a reveal that shell thickness has a noticeable influence on the mechanical behavior of the sintered structures. As the thickness of the silver shell increases, the tensile strength and fracture strain display a non-monotonic variation. Both parameters first increase and then decrease with increasing shell thickness. Among the investigated structures, the Ag6Cu3 configuration demonstrates the most favorable mechanical performance, showing the highest peak stress and a pronounced plastic deformation stage.

When the silver shell is relatively thin, as in the Ag6Cu1 configuration, the protective and diffusion-assisted role of the shell layer is limited, which weakens the interfacial bonding between particles during sintering. On the other hand, when the shell becomes excessively thick, as in the Ag6Cu5 structure, the increased proportion of the shell layer alters the structural balance between the core and shell regions, leading to reduced mechanical strength. These results suggest that an intermediate shell thickness is beneficial for achieving stronger interparticle bonding while maintaining adequate structural stability.

The densification behavior under varying silver shell thicknesses is shown in Figure 9b. All samples undergo rapid densification in the initial stage, followed by a slower approach to a stable state. Among the samples, the Ag6Cu3 configuration reaches both the fastest densification rate and the highest final equilibrium density. This trend can be attributed to the balance between surface and grain boundary diffusion mechanisms. Silver, having a relatively low melting point (961 °C) and high self-diffusion coefficient, facilitates surface diffusion at the sintering temperature. A shell of moderate thickness provides adequate diffusion pathways without weakening the capillary forces that drive densification, leading to an optimal combination of diffusion flux and driving force.

Figure 9c presents the evolution of the normalized structural deviation index (NSDI) for all atoms during sintering. NSDI quantifies deviations from equilibrium atomic arrangements and captures structural relaxation dynamics. The Ag6Cu3 sample exhibits the lowest NSDI at later stages, indicating enhanced atomic ordering and a reduced defect density. This behavior can be interpreted in terms of interface-driven structural evolution: the lattice mismatch at the core–shell interface generates stress, and a moderately thick silver shell enables plastic relaxation that relieves interfacial strain without causing stress concentration due to dislocation accumulation in overly thick shells. When the silver layer reaches an intermediate thickness (~3 nm), the nucleation and glide of mismatch dislocations reach a dynamic balance, producing a metastable configuration with minimal energy.

The mean square displacement (MSD) evolution in Figure 9d illustrates the relationship between atomic mobility and shell thickness. MSD values increase with time, reflecting atomic diffusion kinetics. The Ag6Cu3 sample consistently shows higher MSD values compared with thinner or thicker shells, indicating greater atomic mobility and longer effective diffusion distances. This can be explained by interdiffusion between Ag and Cu: their limited solubility (~4 at.% Ag in Cu at 800 K) creates a concentration-gradient-driven interdiffusion zone. In moderately thick shells, vacancy flux generated by the Kirkendall effect enhances atomic migration. By contrast, thin shells provide insufficient interdiffusion regions, whereas overly thick shells form a continuous silver matrix that limits interfacial diffusion.

In summary, silver shell thickness strongly affects densification, structural ordering, atomic mobility, and the resulting mechanical performance of core–shell nanoparticle sintered bodies. The Ag6Cu3 configuration, with an intermediate shell thickness, achieves a favorable coupling of surface diffusion and interfacial interdiffusion, forming a metastable, low-defect structure. This structural optimization results in the best combination of strength and ductility, offering valuable insights for multiscale design of core–shell nanomaterials and clarifying the microscopic mechanisms by which shell thickness modulates simultaneous strengthening and toughening.

Compared with existing molecular dynamics studies on metallic nanoparticle sintering, the present results demonstrate a consistent trend in which enhanced atomic diffusion and optimized interfacial structure lead to improved mechanical performance. The identified optimal parameter window (e.g., core particle size and shell thickness) aligns with general theoretical predictions of diffusion-controlled densification and interface-driven strengthening. The present study is subject to several inherent limitations, including the use of idealized core–shell nanoparticle models, relatively high strain rates, and simplified interatomic potentials, which may not fully capture the complexity of real experimental systems. In addition, the simulation timescale and system size are constrained, limiting the ability to describe long-term diffusion behavior and large-scale microstructural evolution. Future work will focus on incorporating more realistic material models, extending simulation scales, and establishing closer integration with experimental validation to further improve the reliability and applicability of the findings.

## 4. Conclusions

In this work, molecular dynamics simulations were conducted to investigate the sintering behavior and mechanical performance of Ag@Cu core–shell nanoparticles. The study systematically evaluated the influence of sintering pressure, temperature, particle size, and silver shell thickness, while clarifying the underlying microscopic mechanisms. The key findings can be summarized as follows:

(1) The nanoparticles undergo a structural transition starting from an FCC-dominated initial configuration. Neck formation and the development of HCP regions occur during intermediate stages, ultimately producing a dense and stable structure. The core–shell design effectively guides atomic diffusion, facilitating interfacial bonding and improving structural stability.
(2) Higher sintering pressure promotes densification and strengthens interparticle bonding by increasing particle contact and lowering atomic migration barriers. Pressures in the range of 600–700 MPa result in the best mechanical performance, with both tensile strength and relative density improving as pressure increases.
(3) Elevated temperatures enhance atomic mobility, accelerating neck growth and reducing porosity. The most significant atomic migration occurs at 650–700 K, yielding sintered structures with high structural integrity and enhanced load-bearing capacity.
(4) Particle size plays a critical role in sintering outcomes. Ag6Cu3 nanoparticles strike a balance between high surface energy and structural stability, exhibiting the highest atomic diffusion activity and an optimal combination of strength and ductility. Smaller particles tend to agglomerate unevenly in the early stage, while larger particles suffer from weaker interfacial bonding due to lower effective surface energy.
(5) The thickness of the silver shell shows a clear threshold effect. Ag6Cu3 nanoparticles achieve an effective combination of surface diffusion and interfacial interdiffusion, with enhanced atomic migration through the Kirkendall effect and plastic relaxation of interfacial strain. This leads to a metastable structure with low defect density. Shells that are too thin fail to provide adequate diffusion pathways, whereas excessively thick shells suppress interdiffusion and generate silver-rich defects, both of which reduce mechanical performance.

In conclusion, tuning processing conditions and structural parameters—specifically, a sintering pressure of 600–700 MPa, a temperature of 650–700 K, and Ag6Cu3 core–shell nanoparticles—produces sintered bodies with high densification, strong interfacial bonding, and excellent mechanical properties. These results offer atomistic insights for the design and process optimization of high-performance core–shell nanoparticle solder pastes.

## Figures and Tables

**Table 1:** Number of Atoms and Proportion in Five Copper-Silver Core–Shell Models.

Model Type	Ag6Cu1	Ag6Cu2	Ag6Cu3	Ag6Cu4	Ag6Cu5
Cross-sectional Diagram	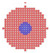	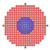	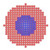	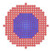	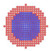
# Number of Total at oms	2147	2183	2255	2339	2495
# Number of Ag atoms	2068	1982	1874	1664	1440
# Number of Cu atoms	79	201	381	675	1055
Ag ratio	96.3%	90.8%	83.1%	71.1%	57.7%

**Table 2:** Sintering and tension process parameters.

Simulation Process	Time (ps)	Temperature (K)		Temperature Change Rate (K/ps)	Pressure (MPa)		Pressure Change Rate (MPa/ps)	Ensemble
		Start	End		Start	End		
① Relaxation	100	300	300	0	0.1	0.1	0	NPT
② T and P rising	300	300	600	1	0.1	500	1.67	NPT
③ T and P holding	300	600	600	0	500	500	0	NPT
④ T and P reducing	300	600	300	1	500	0.1	1.67	NPT
⑤ T and P holding	300	300	300	0	0.1	0.1	0	NPT
⑥ quasi-static tensile	40	300	300	0	0.1	0.1	0	NPT

## Data Availability

The original contributions presented in this study are included in the article. Further inquiries can be directed to the corresponding author.
